# Engineering of Papaya Mosaic Virus (PapMV) Nanoparticles through Fusion of the HA11 Peptide to Several Putative Surface-Exposed Sites

**DOI:** 10.1371/journal.pone.0031925

**Published:** 2012-02-21

**Authors:** Gervais Rioux, Cindy Babin, Nathalie Majeau, Denis Leclerc

**Affiliations:** Infectious Disease Research Centre, Laval University, Quebec City, Quebec, Canada; Centers for Disease Control and Prevention, United States of America

## Abstract

Papaya mosaic virus has been shown to be an efficient adjuvant and vaccine platform in the design and improvement of innovative flu vaccines. So far, all fusions based on the PapMV platform have been located at the C-terminus of the PapMV coat protein. Considering that some epitopes might interfere with the self-assembly of PapMV CP when fused at the C-terminus, we evaluated other possible sites of fusion using the influenza HA11 peptide antigen. Two out of the six new fusion sites tested led to the production of recombinant proteins capable of self assembly into PapMV nanoparticles; the two functional sites are located after amino acids 12 and 187. Immunoprecipitation of each of the successful fusions demonstrated that the HA11 epitope was located at the surface of the nanoparticles. The stability and immunogenicity of the PapMV-HA11 nanoparticles were evaluated, and we could show that there is a direct correlation between the stability of the nanoparticles at 37°C (mammalian body temperature) and the ability of the nanoparticles to trigger an efficient immune response directed towards the HA11 epitope. This strong correlation between nanoparticle stability and immunogenicity in animals suggests that the stability of any nanoparticle harbouring the fusion of a new peptide should be an important criterion in the design of a new vaccine.

## Introduction

Papaya mosaic virus (PapMV) is a member of the large family of *Flexiviridae* in the genus Potexvirus. The virus has a flexuous rod shape of 500 nm in length and 14 nm in diameter made of a single viral capsid protein (CP) and the genomic positive sense RNA [1]. We have previously shown that expression of PapMV CP in bacteria (*E. coli*) leads to production of virus like particles (VLPs) or nanoparticles that can be affinity-purified easily using a 6xH tag [2]. The nanoparticles are non-infectious and are similar in shape and appearance to the wild type virus purified from infected plants [2].

In the last few years, we have shown that PapMV nanoparticles can be used as a vaccine platform via the fusion of a peptide antigen to the C-terminus of the PapMV CP [3], [4], [5], [6]. In fact, the capacity of producing a long-lasting humoral response has been exploited to produce antibodies against a fused HCV immunogenic epitope that was demonstrated to present the peptide in the appropriate conformation [4]. Also, fusion of the universal M2e peptide antigen derived from influenza M2 protein was showed to trigger a protective humoral response against a lethal influenza infection in mice [3]. For each of those fusions and others [7], self-assembly of the recombinant PapMV CP into nanoparticles ranging from 60 to 100 nm in length was shown to be critical to the induction of an efficient humoral response to the fused peptide antigen [3], [4]. We have also shown that PapMV nanoparticles can trigger a cytotoxic (CTL) immune response to a fused CTL epitope through loading of MHC class I and the proliferation of CD8+ human T cells [6]. In another study, fusion of the p33 CTL epitope derived from the lymphocytic choriomeningitis (LCMV) surface glycoprotein to PapMV CP was sufficient to provide complete protection to a LCMV challenge [5].

PapMV nanoparticles can also be used as an adjuvant to larger antigens and proteins. We showed an improved IgG2a response to the *Salmonella typhi* porin OmpC [8], which led to better protection to a challenge with this pathogen. We also demonstrated an improvement in the seasonal flu vaccine using PapMV nanoparticles, with an increased humoral and CTL response to a conserved epitope of the virus leading to protection against a heterosubtypic strain of influenza [9]. Thus, taken together, these results show that PapMV nanoparticles can be used successfully as a vaccine platform [3], [4], [5], [6] and an adjuvant [8], [9] to improve the humoral and CTL response to a given peptide or large protein antigen [5], [6], [9]. Finally, it is also recognized that PapMV nanoparticles are perceived by immune cells as a pathogen-associated pattern (PAMP) [8], [9].

However, while the PapMV vaccine platform is clearly versatile, and has been shown to tolerate the fusion of several peptides to its C-terminus [3], [4], [5], [6], [7], it is also possible that the fusion of certain peptides could interfere with the assembly process of the protein into nanoparticles and consequently affect its ability to trigger a proper immune response. It is therefore of interest to search for other sites of fusion in the PapMV CP to improve this vaccine tool. Considering that fusion might disrupt the nearby structure by modifying the surrounding charge and hydrophobicity of the protein, our goal was to find other sites of fusion in the PapMV CP that can tolerate the fusion of a short epitope: the influenza HA11 epitope. In this study, we tested seven new sites of fusion through insertion of HA11 peptide into PapMV CP and evaluated the capacity of those newly engineered VLPs to trigger a proper immune response to the HA11 antigen.

## Results

### Insertion of the HA11 peptide into 7 putative surface exposed sites of the PapMV-CP

**Figure 1:**
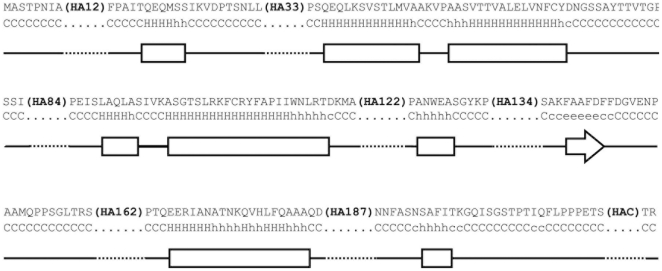
PapMV amino acid sequence and structure. PapMV capsid protein amino acid sequence with each site of fusion and a bioinformatic secondary structure prediction (adapted from Lecours 2006).

Expression of PapMV-CP in *E. coli* leads to the formation of nanoparticles that have a morphology comparable to the wild type virus [2]. Our objective was to evaluate the capacity of the PapMV platform to tolerate fusion of the HA11 peptide leading to the formation of nanoparticles. The HA11 epitope was chosen for fusion given its small length and the availability of commercial tools to study this epitope. A glycine residue was introduced at the N-terminus of the HA11 epitope to disrupt any secondary structure of the PapMV that could be created by the insertion of the HA11 peptide. Fusions were made at 7 specific sites in the capsid protein taking into consideration the bioinformatic prediction of the secondary structure of PapMV from Lecours (2006). Thereby, our attempts at fusions were made in unstructured regions of the PapMV CP located between highly ordered α-helices and β-sheets of the protein after the residues 12, 33, 84, 122, 134, 162, 187, and finally at the extreme C-terminus of the protein as our control point of fusion (Figure 1). A fusion was made after position 12, but not beyond that point, because of the F13 residue in the PapMV N-terminus that was shown previously to play an important role in the self-assembly of PapMV nanoparticles [10].

**Figure 2:**
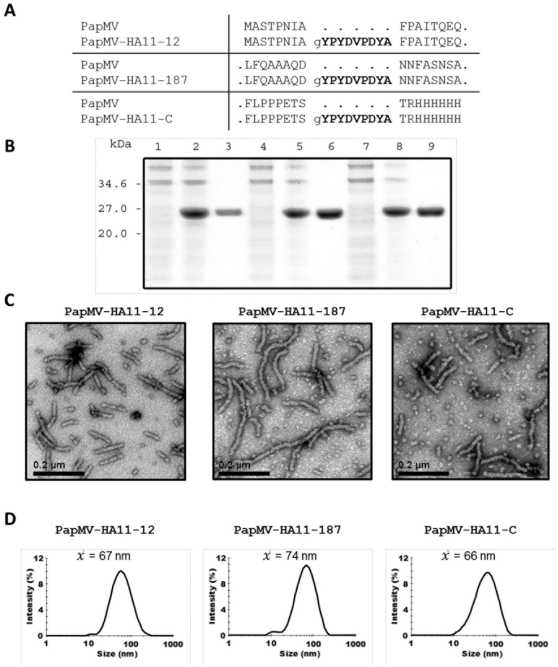
PapMV-HA11 recombinant proteins. The three PapMV-HA11 fusions produced have characteristics similar to those of PapMV nanoparticles. (A) The sequence of the PapMV-CP-HA11 proteins produced. (B) Bacterial lysate of the culture before induction (first lane), after induction with IPTG (second lane), and after successful purification with nickel beads, third lane, of PapMV-CP-HA11-12 (lane 1–3), 187 (lane 4–6) and C (lane7–9). (C)Transmission electron microscope images of each HA11 fusion. (D) Size of VLPs recorded by dynamic light scattering (DLS).

Recombinant proteins harbouring the fusion at position 33, 84, 122, 134 and 162 led to the production of unstable proteins that could not be studied. However, we were able to isolate large amounts of the recombinant proteins harbouring fusions located after the residues 12 and 187 and, as expected, at the C-terminus (Figure 2AB). As observed by TEM, the three recombinant proteins (PapMV-HA11-12, PapMV-HA11-187 and PapMV-HA11-C) could self-assemble into nanoparticles that were similar in appearance to other recombinant nanoparticles reported previously by our group (Figure 2C) [3], [4], [5], [6], [7]. Dynamic light scattering (DLS) revealed that PapMV-HA11-12 and C yielded shorter VLPs with an average size of 67 nm and 66 nm, respectively (Figure 2D). PapMV-HA11-187 had a length of 74 nm. Those lengths are in agreement with the approximate length of PapMV nanoparticles observed by electron microscopy (Figure 2C).

### The peptide HA11 is exposed at the surface of engineered PapMV nanoparticles

**Figure 3:**
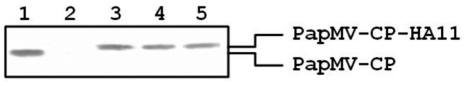
Immunoprecipitation and western blot of PapMV with or without HA11 fusion. PapMV was immunoprecipitated with anti-PapMV mouse serum (lane 1) and with anti-HA11 monoclonal antibody (lane 2). PapMV-HA11-12, 187 and C are immunoprecipitated by anti-HA11 monoclonal antibody (lane 3–5) confirming that the HA11 peptide is at the surface.

To evaluate the surface availability of the HA11 peptide on the recombinant nanoparticles, we performed an immunoprecipitation using an HA11 monoclonal antibody. PapMV nanoparticles without fusions were efficiently recognized by anti-PapMV mouse serum (Figure 3, lane 1) but not by an HA11 specific monoclonal antibody (Figure 3, lane 2). All the PapMV nanoparticles fused to the HA11 epitope were efficiently immunoprecipitated using the monoclonal HA11-specific monoclonal antibody (Figure 3, lane 3–5) suggesting that the HA11 epitope is available at the surface of the recombinant nanoparticles. This suggests that the HA11 epitope has the right conformation and that it is available for interaction with B-lymphocytes.

### Characterization of biochemical and biophysical properties of recombinant PapMV nanoparticles

**Figure 4:**
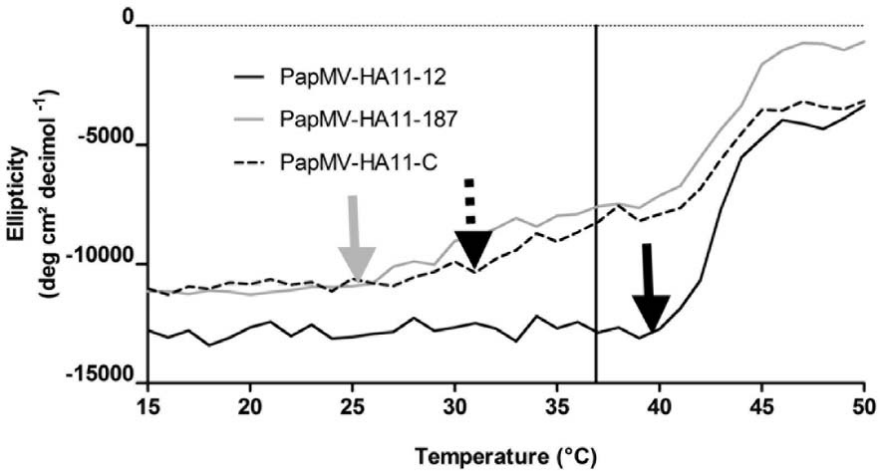
Structural changes in PapMV CP in the different recombinant nanoparticles induced by an increase in temperature. Each of the recombinant nanoparticles (PapMV-HA11-12, PapMV-HA11-187 and PapMV-HA11-C) at a concentration of 0.1 mg/ml were heated in steps of 1°C and secondary structure changes of the protein was monitored by circular dichroism. The read-out was performed at a wave length of 208 nm. The arrows show the point of inflection for each of the nanoparticles. The black bar represents the body temperature of mice (36.9°C).

Each fusion can potentially influence the structure of the PapMV CP comprising the nanoparticles and therefore affect nanoparticle stability. To evaluate the stability of the nanoparticles, we evaluated their ability to tolerate heat. Any conformational change in the nanoparticles was monitored by examining CD spectra (CD) and DLS at increasing temperatures. The CD spectra taken at a wave length of 208 nm—the major absorption point for the PapMV α-helices—revealed that the fusion to position 187 and at the C-terminus were more sensitive to heat since a change in the secondary structure was measured with temperature exceeding 30°C (see dashed arrow in Figure 4). Interestingly, the point of inflection of the PapMV-HA11-12 nanoparticles was found at approximately 40°C (Figure 4; black arrow), and these nanoparticles appear to be more stable than the other 2 recombinant nanoparticles.

**Figure 5:**
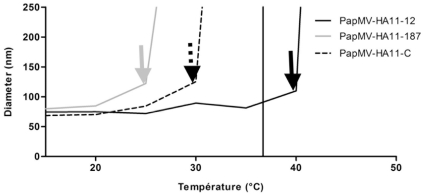
Aggregation of recombinant nanoparticles upon heating. Each of the recombinant nanoparticles at a concentration of 0.1 mg/ml were heated by steps of 5°C and DLS spectra of the samples was measured. The approximate size of the particles measured indicates the level of aggregation of the samples. The arrows show the point of inflection for each of the nanoparticles. The black bar represents the body temperature of mice (36.9°C).

We hypothesized that changes in secondary structure induced by heat will lead to partial denaturation of the PapMV CP, which will consequently expose hydrophobic residues at the surface of the PapMV nanoparticles and lead to aggregation of the VLPs. To validate this hypothesis, we used DLS to measure precisely the aggregation state of the nanoparticles in solution (Figure 5). The three recombinant nanoparticles showed a similar average size of 75, 70 and 80 nm, respectively, for the PapMV-HA11-12, 187 and C-terminus at 20°C. Upon heating, we observed that PapMV-HA11-187 formed large aggregates when the temperature exceeded 25°C. The same phenomenon occurs with the PapMV-HA11-C construct when the temperature exceeded 30°C, and, finally, the PapMV-HA11-12, which was the most stable, showed formation of large aggregates when the temperature exceeded 40°C. These results are consistent with the data obtained with the CD readout and suggest that PapMV-HA11-12 is the most stable VLP of the three recombinant VLPs produced with the HA-11 antigen in fusion.

### Correlation between nanoparticle stability and their immunogenicity

**Figure 6:**
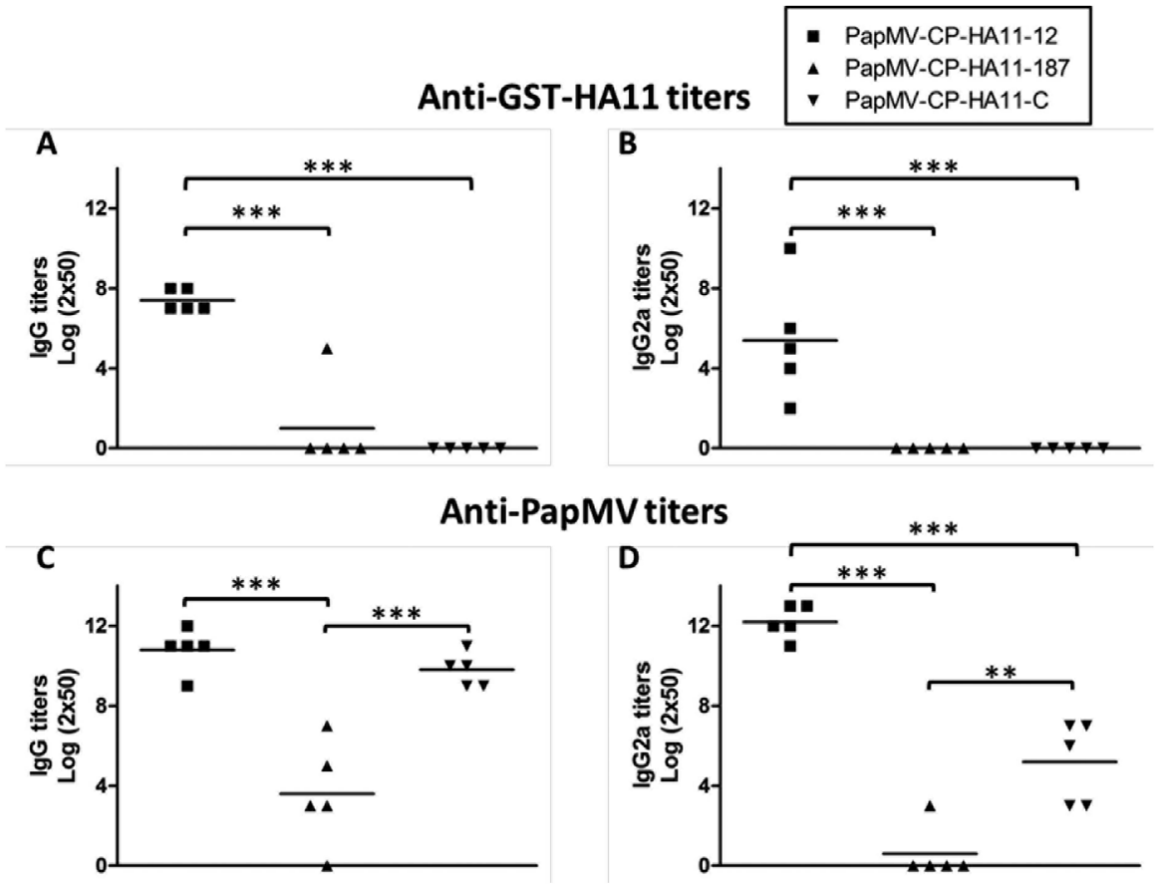
Stable nanoparticles are more immunogenic in animals. Balb/C mice (5 per groups) were immunized twice with a 14-day interval with 100 µg s.c. of PapMV-HA11-12, PapMV-HA11-187 or PapMV-HA11-C, respectively. The total IgG (A) or the IgG2a (B) humoral response directed to the HA11 peptide was measured by ELISA. Also, the total IgG (C) and IgG2a (D) directed to the PapMV CP was measured by ELISA. *** P<0.0001 **P<0.01.

The results described above showed that PapMV CP undergoes conformational changes at elevated temperature that lead to partial denaturation and aggregation of the nanoparticles. PapMV-HA11-187 and C started to aggregate at 25 and 30°C, respectively, i.e., temperatures lower than mouse body temperature (36.9°C). Interestingly, PapMV-HA11-12 remains stable at temperatures exceeding this threshold. To determine the impact of particle aggregation on immunogenicity, we injected Balb/C mice, 5 per group, with 100 µg PapMV nanoparticles of each construction. We evaluated the humoral response triggered against the HA11 peptide (Figure 6 A–B) and to naked PapMV nanoparticles (Figure 6 C–D). Total IgG titres as well as the IgG2a isotype titres were measured. We found that PapMV-HA11-12 nanoparticles were by far the most immunogenic, and the only particles able to trigger a very high humoral response against the HA11 peptide (Figure 6 A–B). PaMV-HA11-187 and C were unable to produce a significant immune response to the HA11 peptide. As expected, the PapMV-HA11-12 construct also showed a significantly higher humoral total IgG response against the PapMV vaccine platform then the PapMV-HA11-187 (Figure 6 C–D). Although total IgG titres of PapMV-HA11-12 and C were similar, PapMV-HA11-12 had a significantly higher IgG2a response against the PapMV platform. PapMV-HA11-C, which was shown to be more stable than PapMV-HA11-187, showed a higher total IgG and IgG2a response to PapMV than PapMV-HA11-187.

## Discussion

Using the HA11 peptide as a model antigen, we tested 8 different sites of fusion on the PapMV CP and evaluated the ability of the resulting constructs to self-assemble into nanoparticles. Only three sites were shown to tolerate the fusion—the others leading to unstable proteins. These three sites were the C-terminus, and positions directly after amino acid 187 and amino acid 12, near the N-terminus. These three different recombinant proteins led to the formation of nanoparticles that present HA11 at their surface.

Our laboratory has previously confirmed on several occasions that the C-terminus of the PapMV CP is located at the surface of the VLPs, and leads to the development of a humoral [3], [4] or cytotoxic immune response [5], [6]. Recently, the C-terminus of potato virus X (PVX) CP, the type member of the potexvirus family, was shown to be exposed at the surface of the virus particle [11]. Therefore, it is reasonable to expect the CP of other members of the same group to tolerate fusion at this position. We showed that it is possible to fuse a peptide at the N-terminus (after amino acid 12) of the PapMV CP and still get self-assembly into VLPs. Fusion at the N-terminus of the PVX CP was also shown to be tolerated and to lead to the formation of virus particles in a plants [11], [12], [13], [14], [15]. In addition, the N-terminus was predicted to be located at the surface of the potato virus X using tritium planigraphy [16], [17]. Another potexvirus, the bamboo mosaic virus (BaMV) was also showed to support substitution of its native N-terminus by a large peptide of 37 amino acids derived from the VP1 protein of foot and mouth disease virus (FMDV) [18]. Our results confirm, and are in agreement with those reports, since we showed that the N-terminus of PapMV coat protein is exposed at the surface and can be used efficiently as a site of fusion for an epitope.

We show here for the first time in the potexvirus group, that it is possible to perform a fusion in the interior of the CP after amino acid 187 and maintain the ability to self-assemble and form VLPs. Nanoparticles harbouring a fusion after amino acid 12, 187 or at the C-terminus were very similar in appearance except that fusions made at the N-terminus and C-terminus appeared to be slightly shorter as compared to other fusions.

Interestingly, nanoparticles harbouring a fusion after amino acid 12 (PapMV-HA11-12) were more stable, and were the only nanoparticles able to trigger an immune response to the HA peptide. It is likely that the CP of the unstable nanoparticles are denaturated at 37°C when injected into animals, which leads to the presentation of hydrophobic residues at the surface of the VLPs that in turn lead to non-specific aggregation of degraded material that is less immunogenic. This result revealed the importance of maintaining the highly repetitive and crystalline structure of the nanoparticles after injection into animals to insure an optimal immune response to the target HA peptide. Our results also suggest that measuring the stability of the nanoparticles using the CD spectrum and DLS is a good way of predicting the ability of a newly engineered VLPs to trigger an efficient immune response to the peptide of interest.

To make the link between the experiments presented in this manuscript and previous work published by our group, we have evaluated the stability of the PapMV-M2e-C construct harbouring the fusion of the M2e peptide (28 a.a.) of influenza virus to the C-terminus of the PapMV CP previously described by our group [3]. We showed that this fusion lead to production of nanoparticles that are unstable at temperature exceeding 30°C (Figure S1-D). This is similar to the PapMV-HA11-C construct that was showed to be not immunogenic (Figure 5). Even if they are unstable at 37°C, the PapMV-M2e-C nanoparticles were shown to trigger a significant immune response to the M2e peptide and trigger protection to an influenza challenge. This response was further improved by addition of PapMV nanoparticles (without fusion) probably because they are more stable and better adjuvant. Based on the present study, it is likely that the of fusion at the C-terminus for this peptide is not optimal.

In an attempt to stabilise the PapMV nanoparticles harbouring the M2e peptide, we have produced the constructs PapMV-M2e-12 (Figure S1-A). We could produce and purify the chimeric protein easily but we were unable to get nanoparticles with this construct. The fusion of the M2e peptide inhibited the self assembly of the protein and lead to production of small aggregates (Figure S1- B). Therefore, we did not pursue with immunization of animals with those proteins.

PapMV nanoparticles are very immunogenic and a strong humoral response is usually directed to the PapMV CP that is the building block of the nanoparticles. We previously showed that pre-existing antibodies directed to PapMV do not affect the ability of the PapMV nanoparticles to boost the humoral response toward the antigen [3], [4], [20]. We have also validated this observation by measuring the antibody response directed to the PapMV CP and the HA11 peptide 14 days after one immunization with the PapMV-HA11-12, PapMV-HA11-187 and PapMV-HA11-C respectively (Figure S2). We showed that only the PapMV-HA11-12 nanoparticles can mount a IgG2a response to HA11 (Fig. S2-B) and a significant IgG and IgG2a response to PapMV (Figure S2-CD). Therefore, even if high titers of antibodies directed to the PapMV are present in the animal when the boost is being administered, the second injection with PapMV-HA11-12 lead to a significant improvement of the immune response directed to the HA11 peptide (Figure 6). Consistent with previous observations, we propose that pre-existing antibodies to the PapMV CP do not affect the ability of PapMV nanoparticles to further boost the immune response to the HA11 peptide.

In conclusion, our data suggest that it is important to evaluate the stability of future fusions to eliminate from screening programs any VLPs that are unstable, and thus less immunogenic, at body temperature. This process would increase the production and study effectiveness of the PapMV as a vaccine platform. This manuscript also reveals the plasticity of the PapMV vaccine platform since three different sites are now available to perform fusions. It is likely that it will be possible to produce stable PapMV nanoparticles through a fusion after amino acid 187 if the context of the fusion is changed or if peptide other than HA11 that is less detrimental to the stability of the nanoparticles is used.

## Materials and Methods

### Production of PapMV nanoparticles

Oligonucleotides used in PCR for the insertion of the HA11 fusion are described in Table 1. The resulting linear vector harbouring the HA11 coding region fused to the PapMV was digested with *Acc651* restriction enzyme and ligated using T4 DNA ligase (New Englands Biolabs). Expression and purification of PapMV nanoparticles fused to the HA11 peptide was performed as described previously [3]. The production of M2e fusion in PapMV was done as described previously [3]. Levels of expression for each recombinant nanoparticle were determined by SDS-PAGE. LPS contamination was always less than 50 endotoxin (EU) units/mg of protein for viable nanoparticles. The size and structure of the nanoparticles were confirmed by observation on a TEM (JEOL -1010, Tokyo, Japan).

**Table 1:** Forward and reverse oligonucleotides used to produce the seven PapMV-HA11 recombinant proteins.

Name	Oligonucleotide sequence
**HA12**	
Forward	5′-ACGT***GGTACC***CGTACGACGTTCCGGATTACGCGTTCCCCGCCATCACCCAGGAAC-3′
Reverse	5′-ACGT***GGTACC***CGGCTATGTTGGGTGTGGATGC-3′
**HA33**	
Forward	5′-ACGT***GGTACC***CGTACGACGTTCCGGATTACGCGCCCTCCCAAGAGCAGTTAAAG-3′
Reverse	5′-ACGT***GGTACC***CCAGAAGATTGGACGTTGGATC-3′
**HA84**	
Forward	5′-ACGT***GGTACC***CGTACGACGTTCCGGATTACGCGCCGGAGATATCACTGGCACAA-3′
Reverse	5′-ACGT***GGTACC***CTATTGATGATGGGCCAGTCAC-3′
**HA122**	
Forward	5′-ACGT***GGTACC***CGTACGACGTTCCGGATTACGCGCCTGCCAATTGGGAGGCCTCA-3′
Reverse	5′-ACGT***GGTACC***CAGCCATTTTGTCCGTCCTCAG-3′
**HA134**	
Forward	5′-ACGT***GGTACC***CGTACGACGTTCCGGATTACGCGAGCGCCAAATTTGCCGCGTTC-3′
Reverse	5′-ACGT***GGTACC***CTGGCTTGTATCCTGAGGCCTC-3′
**HA162**	
Forward	5′-ACGT***GGTACC***CGTACGACGTTCCGGATTACGCGCCGACCCAGGAAGAGCGGATT-3′
Reverse	5′-ACGT***GGTACC***CCGACCTGGTTAGTCCCGAAGG-3′
**HA187**	
Forward	5′-ACGT***GGTACC***CGTACGACGTTCCGGATTACGCGAACAACTTTGCCAGCAACTCC-3′
Reverse	5′-ACGT***GGTACC***CGTCCTGTGCCGCGGCTTGGAA-3′
**HAC**	
Forward	5′-ACGT***GGTACC***CGTACGACGTTCCGGATTACGCGACGCGTCACCATCACCATCAC-3′
Reverse	5′-ACGT***GGTACC***CACTAGTTTCGGGGGGTGGAAG-3′

The sequences in bold and ithalic correspond to the *Acc651* restriction site.

### SDS-PAGE and electroblotting

Samples were mixed with one-third of the final volume of loading buffer containing 5% SDS, 30% glycerol and 0.01% bromophenol blue. SDS-PAGE was performed as described elsewhere [19].

### Immunoprecipitation and western blotting

One microgram of each nanoparticle was immunoprecipitated using monoclonal antibody against the HA11 epitope (Cedarlane, cat# CLH104AP, Burlington, Canada) and mice sera against PapMV at a dilution of 1∶150 in TBS containing 1 µg/ml BSA and incubated at 4°C on a shaking table for 1 hour. Sheep anti-mouse IgG secondary antibodies coupled with magnetic dynabeads (Dynal AS, cat# 112.01D, Invitrogen) were used to link primary antibodies. Western blotting was performed by SDS-PAGE and a semi-dry transfer on nitrocellulose membrane. Membrane were blocked with 5% milk and samples were revealed with anti-PapMV rabbit antibodies and anti-rabbit antibodies coupled with alkaline phosphatase diluted 1∶10 000 in the blocking buffer.

### Dynamic light scattering

The size of nanoparticles was recorded with a ZetaSizer Nano ZS (Malvern, Worcestershire, United Kingdom) at a temperature of 10°C at a concentration of 0.1 mg/ml diluted in PBS 1×. The variation in nanoparticle size induced by temperature variations was measured according to the same experimental conditions.

### Circular dichroism spectroscopy

CD spectra were recorded on a Jasco J-815 (Easton, MD, USA) at temperatures ranging from 15°C to 50°C with 1°C steps. A concentration of 0.1 mg/ml of each protein was read in a 1 mm light path quartz cell at a wavelength of 208 nm. Samples were heated to the target temperature, and held at that temperature for 5 seconds before measuring the ellipticity as calculated elsewhere [19].

### Immunization

Five 6- to 8-week-old BALB/c mice were injected subcutaneously with: (i) 100 µg of PapMV nanoparticles; (ii) 100 µg of PapMV nanoparticles and 5 µg of HA11 peptide; or (iii) 100 µg of PapMV-HA11 nanoparticles of each fusion. A booster shot was given 2 weeks after the first injection and blood samples were obtained 2 weeks after the boost.

### ELISA quantification

ELISA was performed as described elsewhere [9] using GST-HA11 at 1 µg/ml and PapMV-CP nanoparticles at 0.1 µg/ml as bait. GST-HA11 was produced through a C-terminal fusion of the HA11 antigen on the PGEX-2T vector (GE Healthcare) and affinity purified according to standard procedures. Serial dilutions of mice sera were done by two-fold steps starting at 1∶50. Results are expressed as an antibody endpoint titer greater than three-fold OD of the background value consisting of preimmune sera. Data were analyzed with a parametric ANOVA test. Tukey's post tests were used to compare differences in antibody titres among groups of mice. Values of **p<0.01 and ***p<0.0001 were considered statistically significant. Statistical analyses were performed with GraphPad PRISM 5.01.

### Ethics statement

All the work with animals has been done with Institution approved ethics protocol by the ‘Comité de Protection des Animaux - CHUQ (CPA-CHUQ). The approval of this project is found under the authorization number 2010148-1.

## Supporting Information

Figure S1 **Stability of PapMV-M2e-C nanoparticles.** (A) Schematic representation of the fusion made on the PapMV CP with the M2e peptide. (B) Electron microscopy micrographs of PapMV-M2e-12 and (C) PapMV-M2e-C. (D) Aggregation of the PapMV-M2e-C nanoparticles upon heating as measured using dynamic light scattering (DLS). The dotted line represent the body temperature of mice.
(TIF)

Figure S2 **Immune response after one immunization.** Balb/C mice (5 per groups) were immunized once with 100 µg s.c. of PapMV-HA11-12, PapMV-HA11-187 or PapMV-HA11-C, respectively. Sera were harvested 14 days following the first immunization. The total IgG (A) or the IgG2a (B) humoral response directed to the HA11 peptide was measured by ELISA. Also, the total IgG (C) and IgG2a (D) directed to the PapMV CP was measured by ELISA. *** P<0.0001.
(TIF)
