# Supplementary figures and images for: Engineering of Papaya Mosaic Virus (PapMV) Nanoparticles through Fusion of the HA11 Peptide to Several Putative Surface-Exposed Sites
Source: PLoS One. 2012 Feb 21;7(2):e31925. doi: 10.1371/journal.pone.0031925 (PMC3283703; doi:10.1371/journal.pone.0031925)

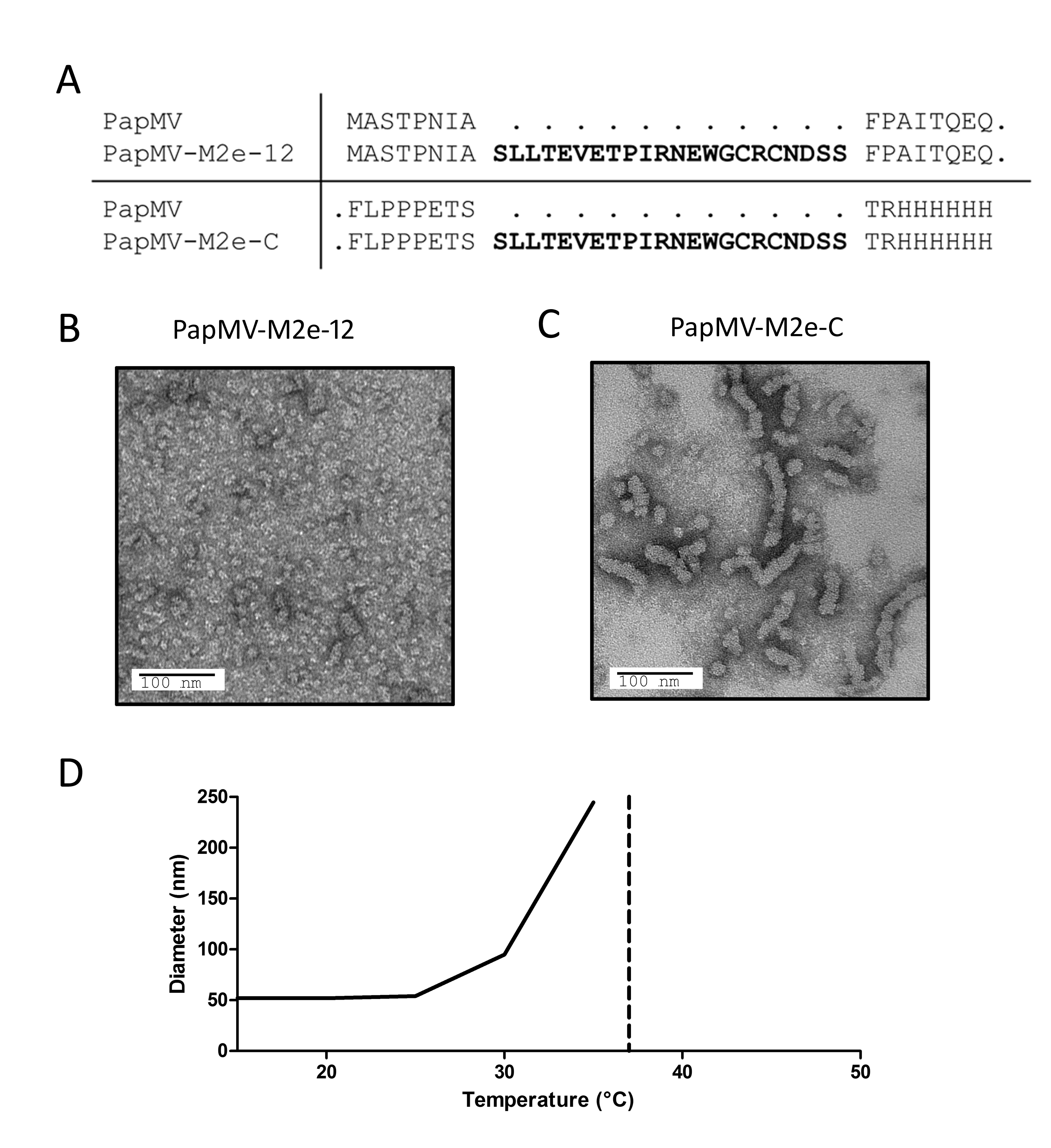

Supplement: Figure S1 — Stability of PapMV-M2e-C nanoparticles. (A) Schematic representation of the fusion made on the PapMV CP with the M2e peptide. (B) Electron microscopy micrographs of PapMV-M2e-12 and (C) PapMV-M2e-C. (D) Aggregation of the PapMV-M2e-C nanoparticles upon heating as measured using dynamic light scattering (DLS). The dotted line represent the body temperature of mice. (TIF) [file pone.0031925.s001.tif]

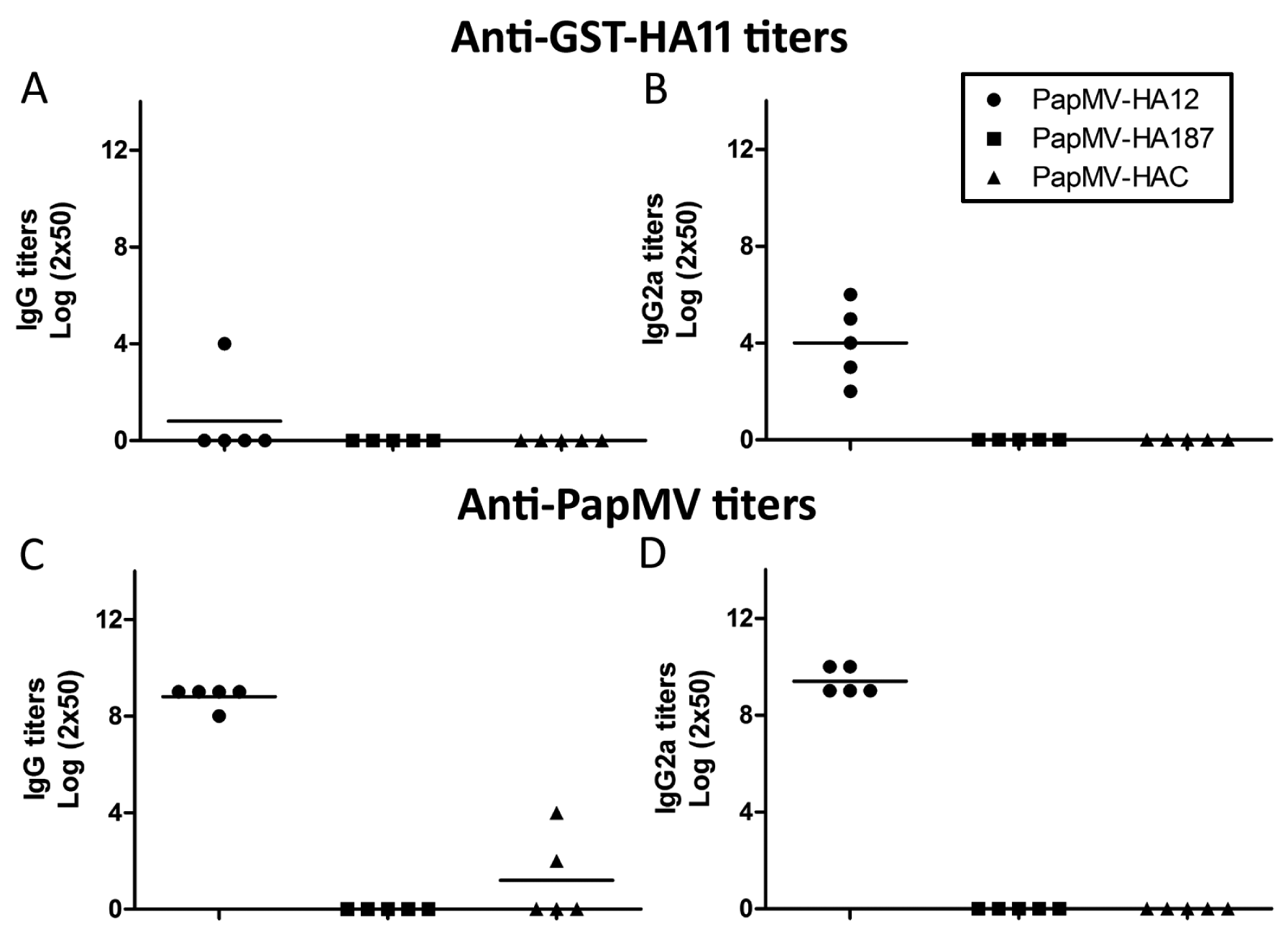

Supplement: Figure S2 — Immune response after one immunization. Balb/C mice (5 per groups) were immunized once with 100 µg s.c. of PapMV-HA11-12, PapMV-HA11-187 or PapMV-HA11-C, respectively. Sera were harvested 14 days following the first immunization. The total IgG (A) or the IgG2a (B) humoral response directed to the HA11 peptide was measured by ELISA. Also, the total IgG (C) and IgG2a (D) directed to the PapMV CP was measured by ELISA. *** P<0.0001. (TIF) [file pone.0031925.s002.tif]
